# Intermittent Starvation Promotes Maturation of Human Embryonic Stem Cell-Derived Cardiomyocytes

**DOI:** 10.3389/fcell.2021.687769

**Published:** 2021-07-30

**Authors:** Jingsi Yang, Nan Ding, Dandan Zhao, Yunsheng Yu, Chunlai Shao, Xuan Ni, Zhen-Ao Zhao, Zhen Li, Jianquan Chen, Zheng Ying, Miao Yu, Wei Lei, Shijun Hu

**Affiliations:** ^1^Department of Cardiovascular Surgery of the First Affiliated Hospital & Institute for Cardiovascular Science, Collaborative Innovation Center of Hematology, State Key Laboratory of Radiation Medicine and Protection, Medical College, Soochow University, Suzhou, China; ^2^Department of Cardiology, The Second Affiliated Hospital of Soochow University, Suzhou, China; ^3^Institute of Microcirculation & Department of Pathophysiology of Basic Medical College, Hebei North University, Zhangjiakou, China; ^4^Center for Molecular Imaging and Nuclear Medicine, State Key Laboratory of Radiation Medicine and Protection, School for Radiological and Interdisciplinary Sciences (RAD-X), Collaborative Innovation Center of Radiation Medicine of Jiangsu Higher Education Institutions, Soochow University, Suzhou, China; ^5^Orthopedic Institute, Medical College, Soochow University, Suzhou, China; ^6^Jiangsu Key Laboratory of Neuropsychiatric Diseases and College of Pharmaceutical Sciences, Soochow University, Suzhou, China

**Keywords:** embryonic stem cells, cardiomyocyte maturation, intermittent starvation, pluripotent stem cells, autophagy

## Abstract

Human pluripotent stem cell-derived cardiomyocytes (hPSC-CMs) represent an infinite cell source for cardiovascular disease modeling, drug screening and cell therapy. Despite extensive efforts, current approaches have failed to generate hPSC-CMs with fully adult-like phenotypes *in vitro*, and the immature properties of hPSC-CMs in structure, metabolism and electrophysiology have long been impeding their basic and clinical applications. The prenatal-to-postnatal transition, accompanied by severe nutrient starvation and autophagosome formation in the heart, is believed to be a critical window for cardiomyocyte maturation. In this study, we developed a new strategy, mimicking the *in vivo* starvation event by Earle’s balanced salt solution (EBSS) treatment, to promote hPSC-CM maturation *in vitro*. We found that EBSS-induced starvation obviously activated autophagy and mitophagy in human embryonic stem cell-derived cardiomyocytes (hESC-CMs). Intermittent starvation, via 2-h EBSS treatment per day for 10 days, significantly promoted the structural, metabolic and electrophysiological maturation of hESC-CMs. Structurally, the EBSS-treated hESC-CMs showed a larger cell size, more organized contractile cytoskeleton, higher ratio of multinucleation, and significantly increased expression of structure makers of cardiomyocytes. Metabolically, EBSS-induced starvation increased the mitochondrial content in hESC-CMs and promoted their capability of oxidative phosphorylation. Functionally, EBSS-induced starvation strengthened electrophysiological maturation, as indicated by the increased action potential duration at 90% and 50% repolarization and the calcium handling capacity. In conclusion, our data indicate that EBSS intermittent starvation is a simple and efficient approach to promote hESC-CM maturation in structure, metabolism and electrophysiology at an affordable time and cost.

## Introduction

Cardiovascular disease has become the number one killer of human health (Tan and Ye, 2018). The most common form of cardiovascular disease is myocardial infarction, accompanied by the cumulative loss of functioning cardiomyocytes due to myocardial ischemia, which eventually leads to heart failure. Due to the limited regenerative capability of adult cardiomyocytes, current strategies, including pharmaceutical treatment and revascularization therapy, are ineffective in preventing disease progression. Meanwhile, the primary human cardiomyocytes are difficult to obtain and maintain *in vitro*, which has long been a major problem for mimicking and investigating human heart diseases. Cardiomyocytes (CMs) derived from human pluripotent stem cells (hPSCs), including embryonic stem cells (hESCs) and induced pluripotent stem cells (hiPSCs), show enormous potential for the development of cardiac disease models, drug discovery and cell therapy (Ye et al., 2015; Gintant et al., 2017; Hu et al., 2018). However, these applications are hampered by the immature phenotypes of hPSC-derived cardiomyocytes (hPSC-CMs) that resemble fetal CMs.

The major hallmarks of cardiomyocyte maturation include maturational hypertrophy, well-formed and organized myofibrils, increased DNA content and mitochondrial density, as well as the ability to perform β-oxidation of fatty acids, functional electrophysiology and calcium handling (Matsui et al., 2007). Many approaches such as long-term culture, biophysical, biochemical, and bioelectrical stimulations, have been developed to enhance the maturity of hPSC-CMs (Hirt et al., 2014; Yang et al., 2014). For instance, we and others have reported that retinoic acid and fatty acid treatment could enhance the structural, metabolic and electrophysiological maturation of hPSC-CMs (Yang et al., 2019; Miao et al., 2020). In addition, tissue engineering, 3D culture and miRNAs were also used to promote hPSC-CMs maturation (Lee et al., 2015; Sun and Nunes, 2017). However, much simpler and more effective approaches are urgently needed to drive hPSC-CM maturation at an affordable time and cost.

The perinatal period has been regarded as a critical window for cardiomyocyte maturation *in vivo*. Instantly, after birth, cardiomyocytes experience a short starvation period, due to sudden interruption of nutrient supply and the switch from placental nutrition to nursing (Fang et al., 2014). This starvation period is accompanied by autophagy in the heart, and the morphologic and metabolic changes in cardiomyocytes. In the fetal stage, cardiomyocytes proliferate sharply and utilize glycolysis as a source of energy. After birth, cardiomyocytes undergo hypertrophic growth and show a metabolic shift from glycolysis to oxidative phosphorylation to produce ATP (Chung et al., 2010; Correia et al., 2017). Mitochondria are the key organelles of energy conversion, metabolism and signal amplification in cardiomyocytes. The metabolic switch of cardiomyocytes is closely related to the state of mitochondria. With the development of heart, mitochondria in cardiomyocytes become more elongated and branched, and establish densely distributed cristae (Martin et al., 2014). The selective autophagy of mitochondria, termed mitophagy, plays an important role in the quality control of mitochondria by removal of damaged or immature mitochondria (Bravo-San Pedro et al., 2017). In this regard, starvation-induced autophagy may promote cardiomyocyte maturation both *in vivo* and *in vitro*.

To verify our hypothesis, we starved hESC-CMs with Earle’s balanced salt solution (EBSS), and investigated the effects of starvation on the maturation of hESC-CMs. We observed a rapid activation of autophagy in hESC-CMs after EBSS treatment and found that the intermittent starvation promotes the structural, metabolic, and electrophysiological maturation of hESC-CMs. In summary, our results indicate that intermittent EBSS starvation provides a new direction for promoting the maturation of cardiomyocytes for better application in disease modeling, drug screening and cell therapy.

## Materials and Methods

### Cell Culture and Cardiomyocyte Differentiation

Experiments with the human embryonic stem cells were approved by the Ethical Committee of Soochow University. The human embryonic stem cell lines H1 and hES3 were maintained in PSCeasy^®^ medium (Cellapy, China) on Matrigel-coated dishes. The hESCs at 80% confluence were passaged using 0.5 mM EDTA (Sigma, United States), and 2 μM thiazovivin (Selleck Chemicals, United States) was supplemented for 24 h after cell passage. Cardiomyocyte differentiation was induced in the chemically defined medium CDM3 which is made of RPMI1640 (Thermo Fisher, United States), bovine serum albumin (Sigma-Aldrich, United States) and L-ascorbic acid 2-phosphate (Sigma-Aldrich, United States) as previously described (Miao et al., 2020). Briefly, hESCs at 95% confluence were first cultured in CDM3 supplemented with 5 μM CHIR99021 (Sigma-Aldrich, United States) for 2 days to induce mesoderm differentiation, and the medium was then refreshed with CDM3. Two days later, cells were cultured in CDM3 supplemented with 2 μM Wnt-C59 (Selleck chemicals, United States). The medium was subsequently changed to CDM3 and refreshed daily. Beating cardiomyocytes derived from hESCs were noted around days 9 to 10. The cardiomyocytes were passaged onto gelatin-coated plates and further purified for 4 days in glucose-free RPMI1640 (Thermo Fisher, United States) supplemented with bovine serum albumin, L-ascorbic acid 2-phosphate and sodium DL-lactate (Sigma-Aldrich, United States).

### Cell Starvation

The purified cardiomyocytes were washed 3 times and incubated with EBSS (Sigma, United States) for the indicated times to induce starvation. Following starvation, cardiomyocytes were maintained in CDM3. To induce intermittent starvation, cardiomyocytes at day 15 were treated with EBSS 2 h per day for 5, 10 or 15 days, respectively.

### Quantitative Real-Time PCR

Total RNAs were isolated from cells using TRIzol Reagent (Sigma-Aldrich, United States) and were reverse-transcribed into cDNA using the Takara PrimeScript^TM^ RT Reagent Kit (Takara, Japan). Quantitative real-time PCR (qPCR) was performed on the Applied Biosystems^®^ StepOnePlus^TM^ Real-Time PCR System (Thermo Fisher, United States). The mRNA expression data were normalized to the expression levels of *18S* rRNA, and gene expression levels were calculated by the method of comparative threshold cycle (2^–Δ^ ^Δ^ ^*Ct*^). All primers used for qPCR are listed in Supplementary Table 1.

### Mitochondrial DNA Content Assay

Genomic and mitochondrial DNA (mtDNA) was extracted using the Genomic DNA Extraction Kit (Tiangen, China). Relative mtDNA content was measured by quantitative real-time PCR. The primers used for the mtDNA content assay are listed in Supplementary Table 1.

### Immunofluorescence Staining

The cells were fixed with 4% paraformaldehyde (Sigma-Aldrich, United States) for 15 min at room temperature (RT), followed by permeabilization with 0.5% Triton X-100 (Sigma-Aldrich, United States) for 15 min. The permeabilized cells were blocked in 5% BSA (Sigma-Aldrich, United States) for 1 h at RT and then incubated at 4°C with the indicated primary antibodies overnight. The next day, the cells were washed with PBS-T (0.1% Tween 20 in PBS) and were incubated with the corresponding fluorescent secondary antibodies for 1 h at RT. Subsequently, the cells were stained with Hoechst 33342 for 15 min at RT. The images were captured under an LSM 880 confocal microscope (Carl Zeiss, Germany), and the ImageJ software was used for further analysis. All antibodies used in this study are listed in Supplementary Table 2.

### Western Blotting

The cells were lysed with pre-cooled RIPA buffer containing the protease inhibitor (Roche, United States), sonicated on ice for 10 s, and then centrifuged (12,000 *g*) for 15 min at 4°C to collect the supernatant. The protein concentration was calculated with a BCA kit (Beyotime, China). The same amount of protein samples was separated by SDS-PAGE, and transferred to a PVDF membrane (EMD Millipore, Germany). Subsequently, the PVDF membrane was successively washed with TBS-T (0.1% Tween 20 in TBS) 3 times, blocked in the 5% solution of skim milk powder, and then incubated with the primary antibodies at 4°C overnight. After washing with TBS-T 3 times, the PVDF membrane was incubated with the corresponding HRP-conjugated secondary antibodies for 1 h at RT. The protein bands were visualized using a Western Blot Detection Kit (Cell Signaling Technology, United States) and further quantified using ImageJ software by normalizing the band intensity of interest to that of GAPDH. Antibodies used for western blotting are listed in Supplementary Table 2.

### Flow Cytometric Analysis

The cells were trypsinized into single cells and successively fixed with 1% PFA for 15 min at RT and 90% cold methanol for 15 min at 4°C. The fixed cells were washed with 0.5% BSA in PBS and subsequently incubated with the primary antibodies diluted in PBS containing 0.5% BSA and 0.1% Triton X-100 for 1 h at RT, followed by incubation with appropriate secondary fluorescent antibodies for 30 min. Finally, the cells were analyzed by a Guava easyCyte^TM^ 8 flow cytometer (EMD Millipore, Germany). Antibodies used for flow cytometric analysis are listed in Supplementary Table 2.

### Mitochondrial Staining

For mitochondrial staining, hESC-CMs cultured on coverslips were incubated with prewarmed medium containing 0.1 μM MitoTracker Red CMXRos (Thermo Fisher, United States) for 20 min. These cells were then stained with TNNT2 antibody, followed by incubation with Alexa Fluor 488-conjugated secondary antibody as shown in Supplementary Table 2. After nuclear staining with Hoechst 33342, cells were visualized under an LSM 880 confocal laser scanning microscope (Carl Zeiss, Germany). For the flow cytometric assay, the hESC-CMs incubated with 0.1 μM MitoTracker Red CMXRos were dissociated into single cells before subsequent TNNT2 and nuclear staining, and finally analyzed with Guava easyCyte 8 (EMD Millipore, Germany).

### Mitochondrial Membrane Potential Assay

Mitochondrial activity was evaluated by using the mitochondrial membrane potential assay kit with JC-1 (Beyotime, China). Briefly, hESC-CMs were washed with D-PBS and incubated with JC-1 staining medium at 37°C for 20 min, followed by washing with JC-1 staining buffer and refreshing with cell culture medium. The cells were then observed under an Olympus DP73 fluorescence microscope (Olympus, Japan) to visualize the green JC-1 monomers (λ ex: 490 nm; λ em: 530 nm) representing predominantly depolarized mitochondria and red JC-1 aggregates (λ ex: 520 nm; λ em: 590 nm) forming in polarized mitochondria. In addition, in the shooting process, we carried out at the same time period and used the same exposure time and parameters to ensure the real changes of mitochondrial membrane potential in each group (Wang et al., 2020). Furthermore, the fluorescence intensity of each group was analyzed by ImageJ software. Carbo -nyl cyanide 4-(trifluoromethoxy) phenylhydrazone (FCCP), a potent oxidative phosphorylation uncoupler, could efficiently eliminate the mitochondrial membrane potential. Cells treated with FCCP (10 μM) were used as a positive control showing the transition of red fluorescence to green fluorescence.

### Patch Clamp for Action Potential Recordings

The cardiomyocytes were seeded on the gelatin-coated 35-mm dishes. After cell adherence for 48 h, spontaneous action potentials (APs) of cardiomyocytes were recorded using the patch clamp system. First, the medium was changed to an external bath solution containing 140 mM NaCl, 4 mM KCl, 1.2 mM CaCl_2_, 1 mM MgCl_2_, 10 mM glucose and HEPES, adjust pH to 7.4 with NaOH. The cells were incubated for 15 min in a cell incubator. The internal solution (115 mM potassium aspartate, 15 mM KCl, 4 mM NaCl, 1 mM MgCl_2_, 5 mM Mg-ATP, 5 mM EGTA, and 5 mM HEPES adjusted pH to 7.2 with NaOH) was added into the electrode tube. Using an Axopath 200B amplifier in the current clamp mode, the spontaneous APs were recorded with a current clamp. Finally, the data were collected and analyzed by pClamp 10 software.

### Calcium Transient

The hESC-CMs growing on gelatin-coated coverslips were subjected to calcium imaging. Briefly, cells were incubated in Tyrode’s solution supplemented with 5 μM Fluo-4 AM (Thermo Fisher, United States) for 30 min at 37°C, followed by 3 washes with prewarmed Tyrode’s solution. After immersion in Tyrode’s solution for 5 min, the spontaneous calcium signaling in hESC-CMs was captured by an LSM 880 confocal laser scanning microscope (Carl Zeiss, Germany) with a 63 × oil immersion objective, using the line-scanning mode with a sampling rate of 1 ms/line. The calcium signals were analyzed to calculate the time to peak (s), Vmax (△F/F0/s) and decay time (s).

### Seahorse XF24 Metabolic Flux Analysis

The cells were seeded onto the gelatin-coated XF24 cell culture microplates (Seahorse Bioscience, United States) at 7.5 × 10^4^ cells/well. After 3 days of culture, the oxygen consumption rate (OCR) or extracellular consumption acid rate (ECAR) was detected using a seahorse XF24 analyzer. When the OCR was detected, 2 μM oligomycin, 1 μM FCCP and 0.5 μM rotenone/antimycin A were sequentially added to the cell culture microplate, and the cardiomyocytes were cultured in base medium that contained the desired substrate. When ECAR was analyzed, 10 mM glucose, 2 μM oligomycin and 50 mM 2-DG were sequentially injected into the special measuring solution containing L-glutamine. The OCR or ECAR was normalized to the protein concentration of cardiomyocytes.

### Statistical Analysis

Comparisons between two groups were analyzed using Student’s *t*-test. Comparisons among multiple groups were analyzed with one-way analysis of variance (ANOVA) with the Bonferroni *post hoc* test. Statistical significance was denoted by *p* ≤ 0.05. All data are presented as the mean ± SEM. All experiments were performed at least three times.

## Results

### EBSS Starvation Induces Autophagy and Benefits H1-Derived Cardiomyocytes

By using the chemically defined protocol presented in Supplementary Figure 1A, we successfully differentiated H1 into spontaneously beating cardiomyocytes (H1-CMs) with a high differentiation efficiency (Supplementary Figure 1B). The differentiated cardiomyocytes were further verified by abundant expression of cardiac markers including *TNNT2*, *MYH6*, *TNNI1*, *TNNI3* and α-actinin (Supplementary Figures 1C–E).

**FIGURE 1:**
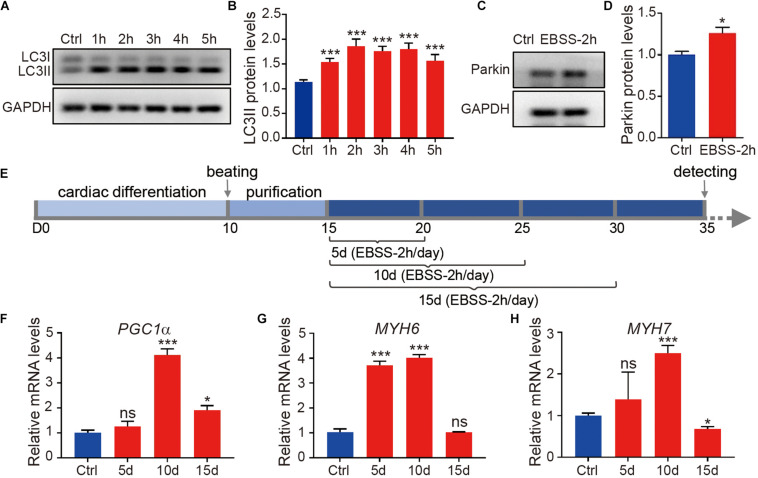
Starvation induces autophagy of human embryonic stem cell-derived cardiomyocytes. **(A)** Western blotting analysis of LC3 expression in H1-CMs immediately after incubation with EBSS for 1 (1 h), 2 (2 h), 3 (3 h), 4 (4 h) and 5 h (5 h), respectively. **(B)** Statistical analysis of penal **(A)**. **(C)** Western blotting analysis of Parkin expression in H1-CMs immediately after EBSS incubation for 2 h. **(D)** Statistical analysis of panel **(C)**. **(E)** Schematic of EBSS-induced starvation (2 h per day) in H1-CMs for 5 (5 day), 10 (10 day) and 15 days (15 day), respectively. **(F–H)** The qPCR analysis of the metabolic marker *PGC1*α and cardiac markers (*MYH6* and *MYH7*) in EBSS-treated H1-CMs on day 35 of cardiomyocyte differentiation. Student’s *t*-test or one-way ANOVA; ^∗^*p* < 0.05, ^∗∗∗^*p* < 0.001, and ns, not significant.

To induce cell starvation, we incubated the H1-CMs with EBSS for 1, 2, 3, 4, and 5 h. Because EBSS starvation is a classic and powerful inducer of autophagy, we first evaluated the autophagy levels in cardiomyocytes immediately after EBSS treatment. As shown in Figures 1A,B, EBSS treatment markedly increased the conversion of microtubule−associated protein light chain 3 (LC3) from LC3-I to LC3-II, an essential event for autophagy activation (Zhu et al., 2007). Our data indicated that EBSS treatments for 1 to 5 h efficiently induced autophagy in H1-CMs, and the 2-h treatment of EBSS was chosen for further studies. We then assessed mitophagy, the mitochondria-specific autophagy, in EBSS-treated cardiomyocytes. The protein expression of the mitophagy marker Parkin was also significantly upregulated in cardiomyocytes by EBSS treatment for 2 h (Figures 1C,D), indicating a successful induction of mitophagy.

To study the effect of starvation on cardiomyocyte maturation, we optimized the time window of EBSS treatment as indicated in Figure 1E. From day 15 of cardiomyocyte differentiation, the H1-CMs were intermittently starved with EBSS (2 h per day) for 5, 10 or 15 days and then maintained in CDM3 until subsequent analysis on day 35. Compared with other groups, H1-CMs intermittently treated with EBSS for 10 days showed the highest upregulation of the mitochondrial and metabolic marker *PGC1*α (Figure 1F), as well as the late cardiac markers *MYH6* (Figure 1G) and *MYH7* (Figure 1H). These data indicated that intermittent starvation for 10 days could efficiently improve cardiomyocyte maturation, which was selected for further analysis.

### Intermittent Starvation Promotes Structural Maturation of hESC-Derived Cardiomyocytes

**FIGURE 2:**
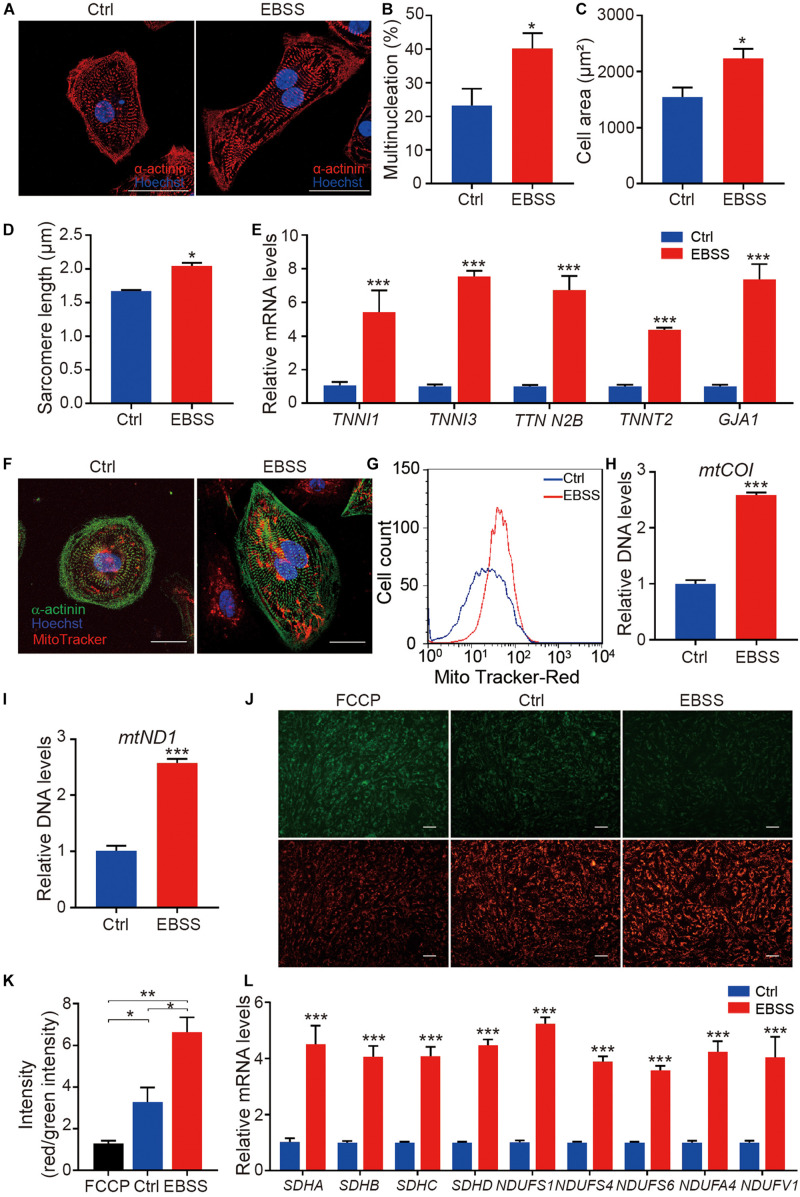
Intermittent starvation promotes structural maturation and mitochondrial maturation of H1-derived cardiomyocytes. **(A)** Representative immunostaining images of α-actinin (red) in control and EBSS-treated H1-CMs. The nuclei were stained with Hoechst 33342 (blue). Scale bar, 50 μm. **(B)** Statistical analysis of multinucleation in the control and EBSS-treated H1-CMs. **(C)** Statistical analysis of cell area in the control and EBSS-treated H1-CMs. **(D)** Statistical analysis of sarcomere length in the control and EBSS-treated H1-CMs. **(E)** The qPCR analysis of cardiac structural genes (*TNNI1*, *TNNI3 TTN N2B*, *TNNT2*, and *GJA1*) in control and EBSS-treated H1-CMs. **(F)** The mitochondria staining with MitoTracker Red CMXRos (red) in the control and EBSS-treated H1-CMs. The cytoskeleton and nuclei were stained with α-actinin antibody (green) and Hoechst 33342 (blue), respectively. Scale bar, 50 μm. **(G)** Flow cytometric analysis showing more abundant mitochondrial content in EBSS-treated H1-CMs. **(H,I)** The qPCR data indicate significantly increased mitochondrial DNA copy number (*mtCO1* and *mtND1* DNA) in EBSS-treated H1-CMs. **(J)** Representative images of mitochondrial membrane potential assay using JC-1 dye in control and EBSS-treated H1-CMs. Green fluorescence signal indicates JC-1 monomers in cells with depolarized mitochondria and red signal indicates JC-1 aggregates forming in polarized mitochondria. Scale bar, 100 μm. **(K)** Quantifications of mitochondrial membrane fluorescence intensity (red/green) in panel **(J)**. **(L)** The qPCR analysis of markers of mitochondrial structure and function. Student’s *t*-test; *p < 0.05, **p < 0.01, ***p < 0.001.

To evaluate the effect of intermittent starvation on cellular morphology and structure, H1-CMs on day 35 were stained with the sarcomeric α-actinin antibody and Hoechst 33342 to visualize the sarcomere structures and nuclei, respectively (Figure 2A). Through statistical analysis, we found intermittent starvation for 10 days obviously increased the ratio of multinucleated cardiomyocytes (Figure 2B), a feature of cardiomyocyte maturation. Compared with the control group, H1-CMs subjected to EBSS-induced starvation exhibited more larger cell area (Figure 2C) and increased sarcomere length (Figure 2D). In addition, qPCR data showed that the mRNA expression of structural maturation-related genes *TNNI1*, *TNNI3, TTN N2B*, *TNNT2*, and *GJA1* were significantly upregulated in H1-CMs after EBSS treatment (Figure 2E). To investigate whether this effect is cell line specific, we further verified the effect of EBSS-induced starvation on the structural maturity in hES3-derived cardiomyocytes (hES3-CMs, Supplementary Figure 2A), as indicated by the increased multinucleation rate (Supplementary Figure 2B) and cell area (Supplementary Figure 2C), the elongated sarcomere (Supplementary Figure 2D), as well as the elevated expression of structural representative markers MYH6 (Supplementary Figure 2E) and MYH7 (Supplementary Figure 2F) and other structural maturation-relative genes (Supplementary Figure 2G). Taken together, these results demonstrated that intermittent starvation promoted the structural maturation of hESC-CMs.

### Intermittent Starvation Enhances the Metabolic Maturation of hESC-Derived Cardiomyocytes

Mitochondrial biogenesis is closely linked with cell metabolism. While cardiomyocytes are more energy-consuming cells that need much more ATP to maintain survival, mitochondrial maturation is very important for the maturation of hESC-CMs. Therefore, we detected the indexes of mitochondrial biogenesis. MitoTracker staining showed a stronger red fluorescence intensity in EBSS-treated H1-CMs, while no obvious effect was detected in mitochondrial distribution by intermittent starvation (Figure 2F). By flow cytometric analysis, we further confirmed the increased fluorescence intensity of MitoTracker in EBSS-treated H1-CMs (Figure 2G). Additionally, as shown in Figures 2H,I, the relative mitochondrial DNA copy numbers, assessed by qPCR detection of the specific mitochondrial DNA regions encoding *mtCO1* and *mtND1*, were significantly increased in EBSS-treated H1-CMs. Meanwhile, we examined the expression of representative marker *PGC1*α (Supplementary Figure 3A) of cardiac metabolism and mitochondrial copy number gene *mtCO1* and *mtND1*(Supplementary Figures 3B,C) in the control and EBSS-treated hES3-CMs, which is consistent with the data obtained in H1-CMs. Therefore, intermittent starvation treatment could increase the mitochondrial DNA content of hESC-CMs.

Mitochondrial membrane potential is an important parameter of mitochondrial activity. We thus analyzed the mitochondrial activity in the control and EBSS-treated H1-CMs by staining with the mitochondrial membrane potential indicator JC-1 and evaluating the resulting red/green fluorescence. After JC-1 incubation, both control and EBSS-treated H1-CMs displayed a strong red JC-1 fluorescence signal, indicating high mitochondrial membrane potential in these cells (Figure 2J). Compared to the control H1-CMs, the ratio of red/green fluorescent intensity was significantly increased in EBSS-treated H1-CMs (Figure 2K). Meanwhile, the qPCR data showed significantly increased expression of genes associated with mitochondrial structure and function, including *SDHA*, *SDHB*, *SDHC*, *SDHD*, *NDUFS1*, *NDUFS4*, *NDUFS6*, *NDUFA4*, and *NDUFV1* in EBSS-treated H1-CMs (Figure 2L).

**FIGURE 3:**
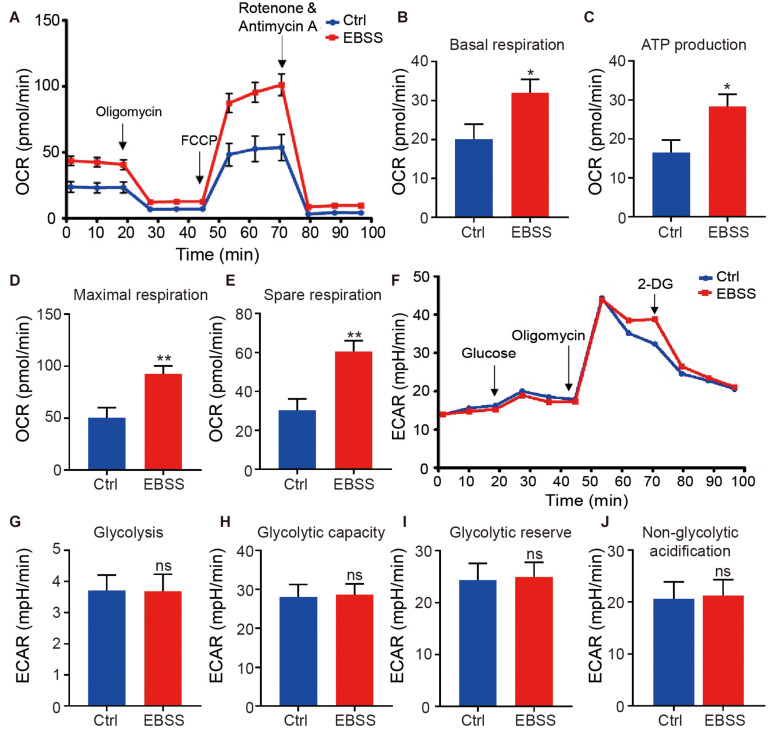
Intermittent starvation enhances metabolic maturation of H1-derived cardiomyocytes. **(A)** Profile of average oxygen consumption rate (OCR) normalized to baseline as evaluated by a Seahorse XF24 Extracellular Flux Analyzer in control and EBSS-treated H1-CMs. **(B–E)** Assessment of basal respiration **(B)**, ATP production **(C)**, maximal respiration **(D)** and spare respiration **(E)** in control and EBSS-treated H1-CMs. **(F)** Representative ECAR traces of control and EBSS-treated H1-CMs obtained to investigate glycolysis using a Seahorse XF24 Extracellular Flux Analyzer. **(G–J)** Quantification of the glycolysis **(G)**, glycolytic capacity **(H)**, glycolytic reserve **(I)** and non-glycolytic acidification **(J)** in control and EBSS-treated H1-CMs. Student’s *t*-test; *p < 0.05, **p < 0.01, and ns, not significant.

The above data indicated the EBSS starvation could promote mitochondrial maturation and increase the mitochondrial activity of hESC-CMs, which is critical for ATP production through oxidative phosphorylation, the major energy source for contraction of the adult heart. We thus further assessed the effect of intermittent starvation on mitochondrial oxidative metabolism in H1-CMs using the XF24 Extracellular Flux Analyzer. We observed a significant increase in the oxygen consumption rate (OCR) of EBSS-treated H1-CMs (Figure 3A). It was mainly reflected in the increased basal respiration, ATP production, maximal respiration and spare respiration (Figures 3B–E). However, no significant change in the extracellular acidification rate (ECAR), an indicator of glycolysis, was observed between the EBSS-treated group and the control group (Figures 3F–J). These results revealed that EBSS-induced starvation could promote the mitochondrial metabolic maturation of H1-CMs.

### Intermittent Starvation Promotes Electrophysiological and Calcium Handling Maturation of hESC-CMs

**FIGURE 4:**
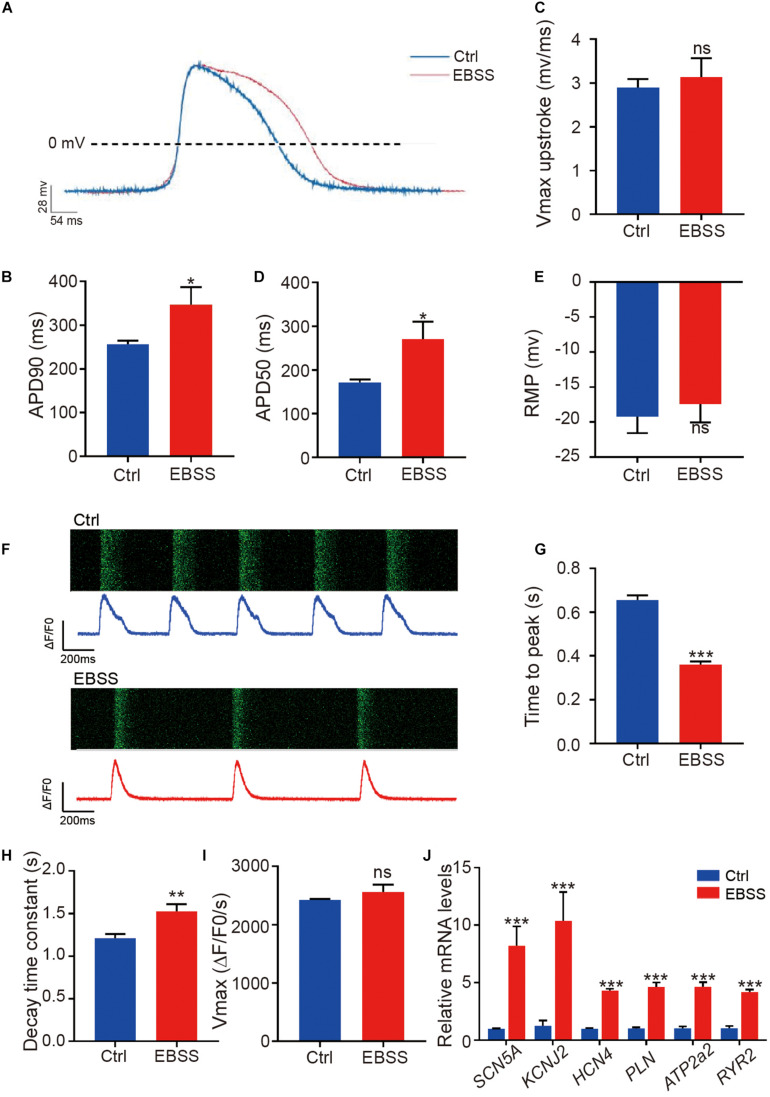
Intermittent starvation promotes electrophysiological maturation and calcium handling of H1-derived cardiomyocytes. **(A)** Representative action potential traces from control and EBSS-treated H1-CMs. **(B–E)** Assessment of APD90 (action potential durations at 90% repolarization), APD50 (action potential durations at 50% repolarization), Vmax upstroke and RMP (resting membrane potentials). **(F)** Representative images showing the time-lapse recording of calcium activity (green) and the calcium handling traces from control (blue trace) and EBSS-treated (red trace) H1-CMs preloaded with the calcium indicator Fluo-4 AM. **(G,H)** Ca^2+^ transient properties of control and EBSS-treated H1-CMs, including the time to peak **(G)**, decay time constant **(H)**, and maximal velocity of upstroke **(I)**. **(J)** The qPCR analysis of electrical conduction (*SCN5A*, *KCNJ2*, and *HCN4*) and calcium handling (*PLN*, *ATP2a2*, and *RYR2*) markers in control and EBSS-treated CMs. Student’s *t*-test; *p < 0.05, **p < 0.01, ***p < 0.001, and ns, not significant.

We further assessed the functional characteristics of H1-CMs including the electrophysiological properties and calcium handling. The patch-clamp technique was used to record the action potential, and the representative spontaneous action potentials from the control and EBSS-treated H1-CMs are clearly shown in Figure 4A. Statistical analysis showed that intermittent starvation significantly prolonged the action potential duration at 90% repolarization (APD90) and action potential duration at 50% repolarization (APD50) in H1-CMs, two parameters usually used to indicate electrophysiological maturation of cardiomyocytes (Figures 4B,C). Nevertheless, we did not observe significant changes in the Vmax upstroke and resting membrane potential (RMP) in the control and EBSS-treated H1-CMs (Figures 4D,E).

Calcium handling is fundamental to cardiac function. In this study, we performed calcium imaging analysis on control and EBSS-treated H1-CMs in which the fluorescent calcium indicator Fluo-4 AM was loaded to detect intracellular free calcium. Compared to the control group, the EBSS-treated cardiomyocytes showed a prolonged calcium transient (Figure 4F), a shortened peak-reaching time (Figure 4G), and a longer decay time (Figure 4H), while no change in Vmax upstroke (Figure 4I) was observed in these cells. The data acquired from hES3-CMs also further validated the accelerative effect of EBSS-induced starvation on cardiac calcium handling (Supplementary Figure 4). Consistent with the prolonged interval of calcium transient interval, we observed a decreased spontaneous contraction rate, another feature of cardiac maturation, in EBSS-treated H1-CMs when compared with that in control cardiomyocytes (Supplementary Movies 1, 2). Meanwhile, the qPCR results demonstrated that the maturation marker genes related to electrical conduction (*SCN5A*, *KCNJ2*, and *HCN4*) and calcium handling (*PLN*, *ATP2a2*, and *RYR2*) were significantly upregulated in H1-CMs by EBSS-induced starvation (Figure 4J). Taken together, these data indicated that the intermittent starvation could facilitate the functional maturation of H1-CMs, including the electrophysiological properties and calcium handling.

## Discussion

Pluripotent stem cell-derived cardiomyocytes are a promising cell resource for cardiac disease modeling, drug screening and cell therapy (van Laake et al., 2008; Hartman et al., 2016). However, hPSC-CMs display immature structural, metabolic and electrophysiological features resembling embryonic or fetal cardiomyocytes, which severely obstruct accurate disease modeling and clinical applications. Prolonged culture (>90 days) has showed some positive effects on cardiomyocyte maturation; however, it is time-consuming and costly. Although a large number of studies have tried to improve the maturation of hPSC-CMs within a reasonable time frame by chemical, genetic, and biomechanical approaches, yet these approaches are not enough to efficiently mature these cells (Karbassi et al., 2020). In this study, we established a novel and accessible approach to promote the structural, metabolic and electrophysiological maturation of hPSC-CMs by EBSS-induced intermittent starvation. This approach is accompanied by autophagy and mitophagy in hPSC-CMs.

Despite a lack of knowledge regarding the regulatory mechanism, the prenatal-to-postnatal transition is believed to present a critical window for cardiomyocyte maturation (Neary et al., 2014; Gentillon et al., 2019). Immediately after birth, neonates sustain severe nutrient starvation and display massive autophagosome formation in their hearts, indicating a critical role of autophagy during heart development (Correia et al., 2017; Kretschmar et al., 2019). Our approach in this study is to mimic the *in vivo* starvation event through EBSS treatment to promote hESC-CM maturation. EBSS is a widely used inducer of autophagy (Bravo-San Pedro et al., 2017; Cai et al., 2019). We also observed dramatic induction of autophagy and mitophagy in EBSS-treated hESC-CMs, which displayed more mature features on day 35 of cardiomyocyte differentiation. Thus, we believe that starvation-induced autophagy is involved in cardiomyocyte maturation *in vitro*. As a highly conserved cellular mechanism of protein recycling, autophagy is adaptively activated by nutrient deprivation, hypoxia, or other stress (Zhu et al., 2007). Numerous molecular mechanisms have been causally implicated in stress-induced autophagy. The nutrient sensors, sirtuin-1 (SIRT1) and adenosine monophosphate-activated protein kinase (AMPK) were recently shown to augment autophagy in cardiomyocytes, as master regulators of hundreds of genes and proteins (Packer, 2020). For instance, AMPK is activated in response to decreased level of ATP/ADP, and inhibits mTOR activity by phosphorylating the tuberous sclerosis complex 2 (TSC2), thereby stimulates autophagy (Matsui et al., 2007). While phosphorylation of AMPK promotes maturation of autophagosomes (Jang et al., 2018), SIRT1 primarily promotes selective clearance of damaged mitochondria (Fang et al., 2014). According to previous studies, we speculated that both AMPK2 and SIRT1 might mediate the effects of EBSS-induced starvation on cardiomyocyte maturation.

Previous studies indicated that the maturation process of hPSC-CMs is stagnated at days 20 – 30 of cardiomyocyte differentiation, when these hPSC-CMs reach the late embryonic stage rather than the neonatal stage (Lee et al., 2010; Uosaki et al., 2015). Thus, we preferred to induce cardiomyocyte starvation before maturation arrest. Through optimizing the time window, we found that intermittent starvation (a 2-h treatment of EBSS per day from day 15) for 10 days is more efficient in promoting the maturation of hESC-CMs. Our study also supports the existence of a critical window for promoting maturation in hPSC-CMs.

Cardiomyocytes undergo dramatic changes in structure, metabolism and function during the course of maturation *in vivo* (Mordwinkin et al., 2013). Notably, our results revealed that EBSS-induced starvation could promote the maturation of hESC-CMs, which was mainly reflected in the following three aspects. Structurally, the EBSS-treated hESC-CMs showed a larger cell size, more organized contractile cytoskeleton and a higher percentage of multinucleate cardiomyocytes. Metabolically, EBSS-induced starvation promoted oxidative phosphorylation. Functionally, EBSS-induced starvation strengthened electrophysiological maturation and the capacity of calcium handling. However, cardiomyocyte maturation is a complex process, involving a series of critical events (Fang et al., 2014; Chen et al., 2019). Despite of partial maturation, EBSS-treated hESC-CMs could not completely mimic adult cardiomyocytes. Thus, combinatorial approaches might be more efficient and necessary to accurately control the maturation of hPSC-CMs to meet different requirements in disease-modeling and clinical applications. Our recent study has indicated that retinoid acid (RA) treatment could also enhance hPSC-CM maturation (Miao et al., 2020), and in the future the combined treatment is expected to further optimize the protocol for hPSC-CM maturation.

In summary, EBSS-induced intermittent starvation is an efficient strategy to promote the structural, metabolic and functional maturation of hPSC-CMs at an affordable time and cost. This new approach will help to improve the applications in cardiac disease modeling, drug screening, and cell therapy.

## Data Availability Statement

The original contributions presented in the study are included in the article/Supplementary Material, further inquiries can be directed to the corresponding authors.

## Author Contributions

SH, WL, MY, ZY, and Z-AZ conceptualized and designed the study. SH, WL, MY, and JY wrote the manuscript. JY, ND, YY, CS, DZ, and XN acquired and analyzed the majority of the data. SH, WL, ZL, JC, and MY revised the manuscript critically for important intellectual content. SH supervised the study and provided main funding support. All authors contributed to the article and approved the submitted version.

## Conflict of Interest

The authors declare that the research was conducted in the absence of any commercial or financial relationships that could be construed as a potential conflict of interest.

## Publisher’s Note

All claims expressed in this article are solely those of the authors and do not necessarily represent those of their affiliated organizations, or those of the publisher, the editors and the reviewers. Any product that may be evaluated in this article, or claim that may be made by its manufacturer, is not guaranteed or endorsed by the publisher.
