# Supplementary material for: Intermittent Starvation Promotes Maturation of Human Embryonic Stem Cell-Derived Cardiomyocytes
Source: Front Cell Dev Biol. 2021 Jul 30;9:687769. doi: 10.3389/fcell.2021.687769 (PMC8362881; doi:10.3389/fcell.2021.687769)
Supplement: Supplementary file 1 [file Data_Sheet_1.pdf]

SUPPLEMENTAL INFORMATION

Intermittent starvation promotes maturation of human embryonic stem cell-derived cardiomyocytes

Jingsi Yang[1,#], Nan Ding [1,#], Dandan Zhao [1,#], Yunsheng Yu [1,#], Chunlai Shao [2], Xuan Ni [1], Zhen-Ao Zhao[3], Zhen Li [4], Jianquan Chen [5], Zheng Ying [6], Miao Yu[1,*], Wei Lei[1,*], Shijun Hu[1,*]

[1] Department of Cardiovascular Surgery of the First Affiliated Hospital & Institute for Cardiovascular Science, Collaborative Innovation Center of Hematology, State Key Laboratory of Radiation Medicine and Protection, Medical College, Soochow University, Suzhou 215000, China; [2] Department of Cardiology, The Second Affiliated Hospital of Soochow University, Suzhou 215004, China; [3] Institute of Microcirculation & Department of Pathophysiology of Basic Medical College, Hebei North University, Zhangjiakou 075000, China; [4] Center for Molecular Imaging and Nuclear Medicine, State Key Laboratory of Radiation Medicine and Protection, School for Radiological and Interdisciplinary Sciences (RAD-X), Soochow University, Collaborative Innovation Center of Radiation Medicine of Jiangsu Higher Education Institutions, Suzhou 215123, China; [5] Orthopedic Institute, Medical College, Soochow University, Suzhou 215007, China; [6] Jiangsu Key Laboratory of Neuropsychiatric Diseases and College of Pharmaceutical Sciences, Soochow University, Suzhou 215123, China.

[#] These authors contributed equally to this work

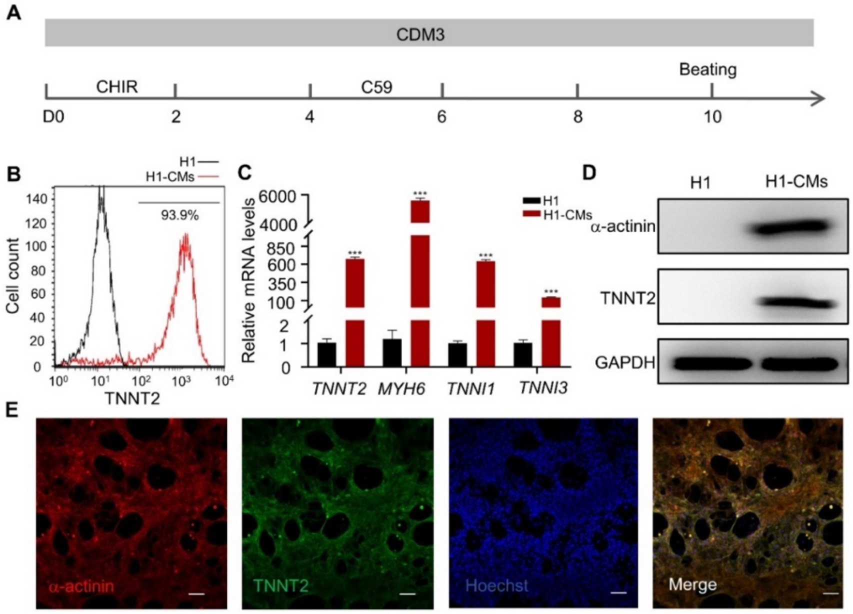

Supplemental Figure 1. Acquisition and identification of H1-CMs. (A) Schematic of cardiomyocyte differentiation protocol. (B) Flow cytometry analysis for cardiac troponin T (TNNT2) to indicate cardiomyocyte differentiation efficiency. (C) qPCR analysis of cardiac marker genes (*TNNT2*, *MYH6*, *TNNI1* and *TNNI3*) in H1-CMs. (D) Western blotting analysis of α-actinin and TNNT2. (E) Immunofluorescent staining of α-actinin (red) and TNNT2 (green) in H1-CMs. Nuclei were stained with Hoechst 33342 (blue). Scale bar, 100 μm. All data are presented as mean ± SEM; Student's *t*-test; ***$p <$ 0.001.

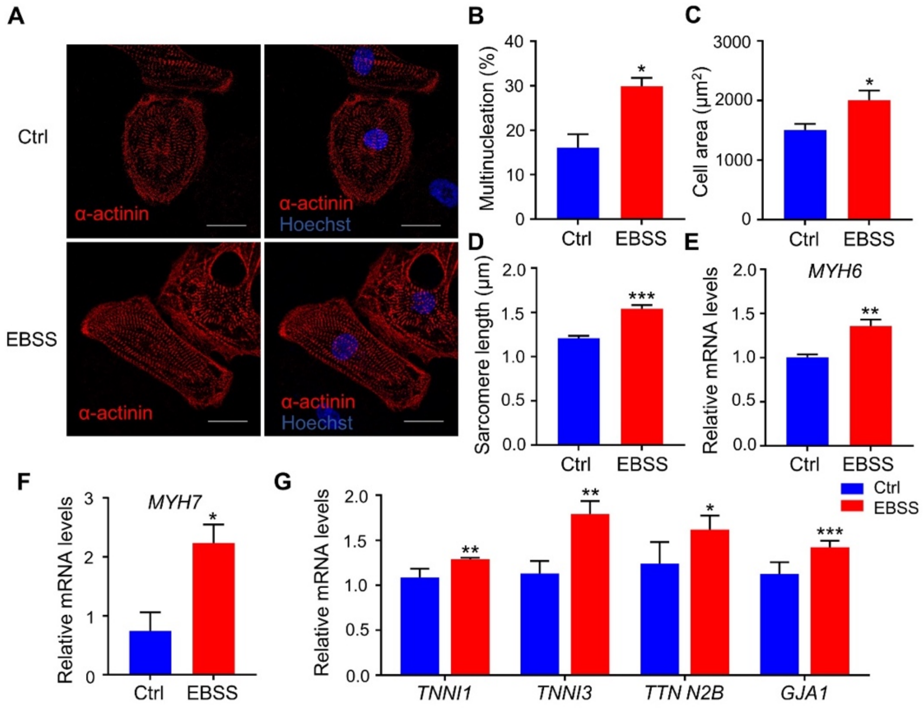

Supplemental Figure 2. Intermittent starvation promotes hES3-CMs structural maturation. (A) Representative immunostaining images of α-actinin (red) in control and EBSS-treated hES3-CMs. The nuclei were stained with Hoechst 33342 (blue). Scale bar, 50 μm. (B) Statistical analysis of multinucleation in the control and EBSS-treated hES3-CMs. (C) Statistical analysis of cell area in the control and EBSS-treated hES3-CMs. (D) Statistical analysis of sarcomere length in the control and EBSS-treated hES3-CMs. (E-F) Representative marker (*MYH6* and *MYH7*) of myocardial structure were increased in EBSS-treated CMs. (G) The qPCR analysis of the structural gene marker *TNNI1, TNNI3, TTN N2B, GJA1*. All data are presented as mean ± SEM; Student's *t*-test; $^{*}p < 0.05$, $^{**}p < 0.01$, $^{***}p < 0.001$.

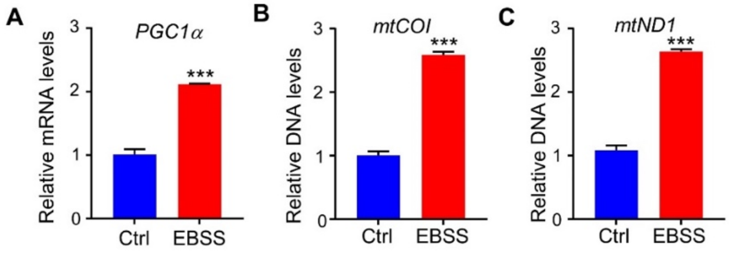

Supplemental Figure 3. Metabolism and mitochondria-related gene expression in hES3-CMs. (A) qPCR analysis of the metabolic marker (*PGC1α*). (B-C) qPCR analysis of the mitochondrial DNA copy number (*mtCO1* and *mtND1* DNA) in EBSS-treated hES3-CMs. All data are presented as mean ± SEM; Student's *t*-test; $^{***}p < 0.001$.

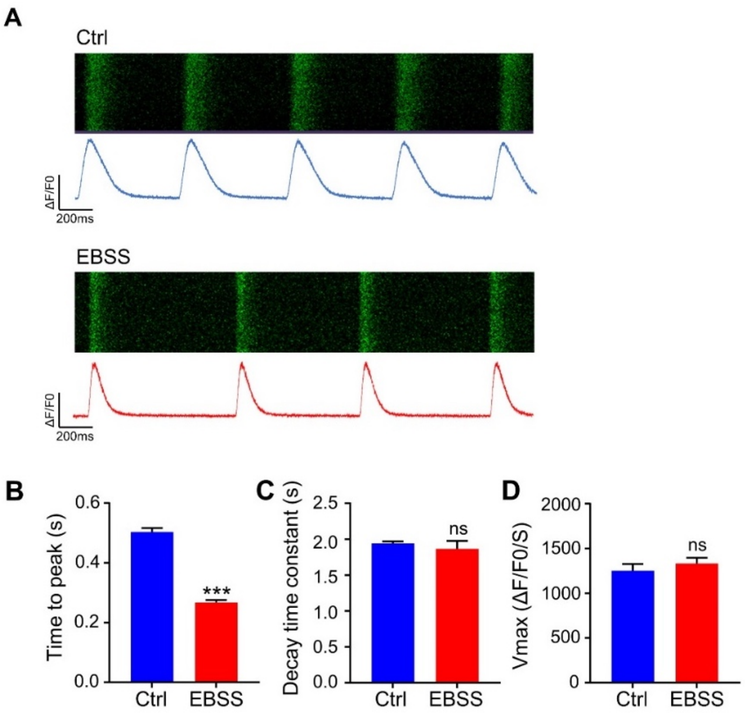

Supplemental Figure 4. Intermittent starvation promotes calcium handling of hES3-CMs. (A) Representative images showing the time-lapse recording of calcium activity (green) and the calcium handling traces from control (blue trace) and EBSS-treated (red trace) hES3-CMs preloaded with the calcium indicator Fluo-4 AM. (B-D) Ca^{2+} transient properties of control and EBSS-treated hES3-CMs, including the time to peak (B), decay time constant (C), and maximal velocity of upstroke (D). All data are presented as mean ± SEM; Student's *t*-test; $^{***}p < 0.001$, and ns, not significant.

Supplemental movie 1. Representative movie of beating H1-CMs.

Supplemental movie 2. Representative movie of beating H1-CMs treated with EBSS.

Supplemental table 1. List for PCR primers.

Genes	Species	Sense (5’→3’)	Antisense (5’→3’)
TNNT2	Human	ACCAAAGCCCAGGTCGTTC	CAGCGCCTGCAACTCATTC
TNNI3	Human	CCTCCAACTACCGCGCTTAT	CTGCAATTTTCTCGAGGCGG
TNNI1	Human	GCTCCACGAGGACTGAACAA	CTTCAGCAAGAGTTTGCGGG
TTN N2B	Human	CCAATGAGTATGGCAGTGTCA	TACGTTCCGGAAGTAATTTGC
MYH6	Human	CAAGAGCCGTGACATTGGTG	AGGTTGGCAAGAGTGAGGTT
MYH7	Human	ACCTGTCCAAGTTCCGCAAG	TCATTCAAGCCCTTCGTGCC
PGC1α	Human	AACAGCAAAAGCCACAAAGACG	GGGGTCAGAGGAAGAGATAAAGTTG
GJA1	Human	CATTGTGGACCAGCGACCTT	GGTGCACTTTCTACAGCACCT
RYR2	Human	TCCGGAAACAGTATGAAGACCA	CACACAACGCTGGCAATTCA
HCN4	Human	AAGCTGCGTTTCGAGGTCTT	GTCTCCTTGTTGCCCTTGGT
ATP2A2	Human	TCAAGCACACTGATCCCGTC	GCTACCACCACTCCCATAGC
KCNJ2	Human	TACGGCGAGAGTCGGAGAT	TGCTTGACCCATCTTGACCAG
PLN	Human	CCCCAGCTAAACACCCGTAA	AGCTGGCAGCCAAATATGAGA
SCN5A	Human	CACGCGTTCACTTTCCTTCG	TCGAAGAGCCGACAAATTGC
SDHA	Human	ACTGTTGCAGCACAGCTAGAA	GCTCTGTCCACCAAATGCAC
SDHB	Human	ACAAGGCTGGAGACAAACCT	CCTTCGGGTGCAAGCTAGAG
18S rRNA	Human	GTAACCCGTTGAACCCCATT	CCATCCAATCGGTAGTAGCG
mtND1	Human	AACCTCAACCTAGGCCTCCT	GAGTTTGATGCTCACCCTGA
mtCOI	Human	ACGTTGTAGCCCACTTCCAC	CATCGGGGTAGTCCGAGTAA
18S rDNA	Human	GCTGAGAAGACGGTCGAACT	CGCAGGTTCACCTACGGAAA

Supplemental table 2. Antibodies used for immunofluorescence (IF), flow cytometry (FAC) & western blotting (WB).

Antibody	Company	Catalog Number	IF	FAC	WB
Troponin T (TNNT2)	Thermo Scientific	MS-295-P1		1:200	1:1000
Troponin T (TNNT2)	Proteintech	15513-1-AP	1:200		
Sarcomeric Alpha Actinin (α-actinin)	Abcam	ab9465	1:200		1:1000
GAPDH	Sungene Biotech	KM9002T			1:1000
LC3I/II	Cell Signaling Technology	4108S			1:1000
Parkin	Cell Signaling Technology	4211S			1:1000
Alexa Fluor® 488 AffiniPure Donkey Anti-Rabbit IgG (H+L)	Jackson ImmuoResearch	711-545-152	1:1000		
Alexa Fluor® 488 AffiniPure Donkey Anti-Mouse IgG (H+L)	Jackson ImmuoResearch	715-545-151	1:1000		
Alexa Fluor® 594 AffiniPure Donkey Anti-Rabbit IgG (H+L)	Jackson ImmuoResearch	711-585-152	1:1000		
Alexa Fluor® 594 AffiniPure Donkey Anti-Mouse IgG (H+L)	Jackson ImmuoResearch	715-585-151	1:1000		
Peroxidase AffiniPure Donkey Anti-Mouse IgG (H+L)	Jackson ImmuoResearch	715-035-151			1:2000
Peroxidase AffiniPure Rabbit Anti-Goat IgG (H+L)	Jackson ImmuoResearch	305-035-003			1:2000
Peroxidase AffiniPure Donkey Anti-Rabbit IgG (H+L)	Jackson ImmuoResearch	711-035-152			1:2000
